# Hybrid Titanium/Biodegradable Polymer Implants with an Hierarchical Pore Structure as a Means to Control Selective Cell Movement

**DOI:** 10.1371/journal.pone.0020480

**Published:** 2011-05-26

**Authors:** Nihal Engin Vrana, Agnès Dupret, Christelle Coraux, Dominique Vautier, Christian Debry, Philippe Lavalle

**Affiliations:** 1 Institut National de la Santé et de la Recherche Médicale, INSERM Unité 977, Strasbourg, France; 2 Faculté de Chirurgie Dentaire, Université Louis Pasteur, Strasbourg, France; 3 Hôpitaux Universitaires de Strasbourg, Hôpital de Hautepierre, Service Otorhinolaryngologie & Chirurgie Cervicofaciale, Strasbourg, France; 4 Institut National de la Santé et de la Recherche Médicale, INSERM Unité 903, Reims, France; Université de Technologie de Compiègne, France

## Abstract

In order to improve implant success rate, it is important to enhance their responsiveness to the prevailing conditions following implantation. Uncontrolled movement of inflammatory cells and fibroblasts is one of these in vivo problems and the porosity properties of the implant have a strong effect on these. Here, we describe a hybrid system composed of a macroporous titanium structure filled with a microporous biodegradable polymer. This polymer matrix has a distinct porosity gradient to accommodate different cell types (fibroblasts and epithelial cells). The main clinical application of this system will be the prevention of restenosis due to excessive fibroblast migration and proliferation in the case of tracheal implants.

**Methodology/Principal Findings:**

A microbead-based titanium template was filled with a porous Poly (L-lactic acid) (PLLA) body by freeze-extraction method. A distinct porosity difference was obtained between the inner and outer surfaces of the implant as characterized by image analysis and Mercury porosimetry (9.8±2.2 µm vs. 36.7±11.4 µm, p≤0.05). On top, a thin PLLA film was added to optimize the growth of epithelial cells, which was confirmed by using human respiratory epithelial cells. To check the control of fibroblast movement, PKH26 labeled fibroblasts were seeded onto Titanium and Titanium/PLLA implants. The cell movement was quantified by confocal microscopy: in one week cells moved deeper in Ti samples compared to Ti/PLLA.

**Conclusions:**

In vitro experiments showed that this new implant is effective for guiding different kind of cells it will contact upon implantation. Overall, this system would enable spatial and temporal control over cell migration by a gradient ranging from macroporosity to nanoporosity within a tracheal implant. Moreover, mechanical properties will be dependent mainly on the titanium frame. This will make it possible to create a polymeric environment which is suitable for cells without the need to meet mechanical requirements with the polymeric structure.

## Introduction

Cell containing biomedical devices such as biosensors, tissue engineering products or cellularized implants must be able to segregate cells they enclose and control their behaviour [1], [2]. This is especially important when the target area's functionality depends on two or more types of cells which are present in a certain orientation. For example, for proper functioning of a tracheal implant, a respiratory epithelium lining on its lumen is essential. This necessitates a smooth implant surface with a porosity smaller than cell size that would allow the migration of epithelial cells from anastomosis sites [3], [4]. On the other hand, if a biodegradable material is used, the bulk of the implant should be populated with connective tissue via migration before extensive degradation, to prevent the mechanical failure or collapse of airway upon implantation. Incorporation of the necessary properties for accommodating these two requirements is challenging.

The primary design concern for implants is their responsiveness to the cell types which are necessary for the functionality of the target tissue. However, inflammation process and movement of fibroblasts during the healing process, which are common themes of mammalian response to foreign bodies, must also be taken into account [5]. Implant designs which would direct and control these body reactions would provide the functional parts of the implant with a better environment for healing.

**Figure 1:**
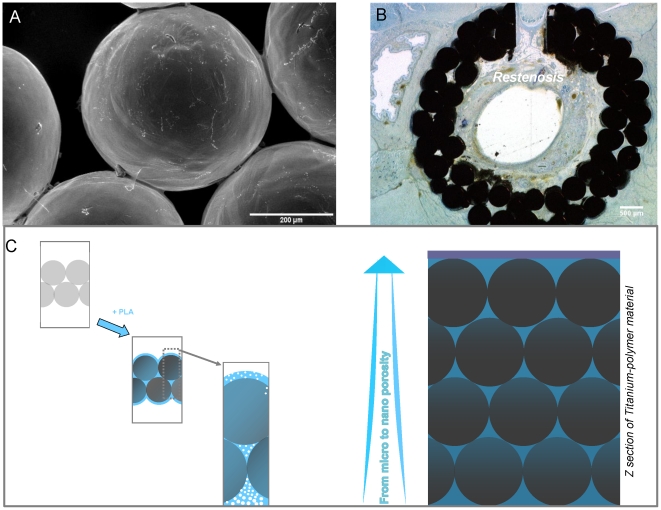
Occurrence of restenosis and proposed solution. a) SEM observation displaying microscopic structure of macroporous titanium implants. b) When implanted(with a slice of native trachea left in place, as seen as a hole), tubular macroporous titanium implants are prone to development of restenosis in rats after 1 month of implantation and subsequent decrease in lumen diameter due to the fact that the open porous structure permits excessive movement of fibroblasts and inflammatory cells as observed by histological sections. c) The scheme of proposed hybrid material to control cell movement in both radial (fibroblasts) and longitidunal (epithelial cells) directions.

Microbead based porous titanium technology allows to create open macroporous structures by using medical grade titanium microbeads. This system is in clinical trial stage in several implant related technologies. Our current efforts are concentrated on development of tracheal, dental, and bone implants by using this technology [6], [7], [8]. The open pore structure of the porous titanium together with its mechanical properties and biocompatibility makes it an ideal implant material for tracheal replacement. The previous efforts in tissue engineering of trachea generally faced up difficulties due to inferior mechanical properties and inflammatory reactions. Moreover most surfaces such as silicone are not good for epithelial cell growth and also prevent vascularization which decreases the functionality of the overall implant [9], [10]. Our previous in vivo experiments with rats and sheep [7], [11] gave promising results but the recurring problem of restenosis, which is the overgrowth of connective tissue, needs to be solved to ensure long-term success of the implant (Figure 1). Extensive restenosis within the lumen must be prevented since it decreases the size of the lumen leading to airway obstruction. For this aim we propose development of a new titanium/biodegradable polymer hybrid material, which can be described as a tissue engineering scaffold within a mechanically stable titanium template. By this method the formation of new diverse tissue can be limited to certain areas of the implant. Such strategy is more appropriate than a total renewal that would compromise mechanical stability since degradation can outpace tissue formation. Moreover, competition for a certain area of the implant such as the lumen between different growing tissues, that results most of the time in population by only one invasive cell type such as fibroblasts, will be controlled [12].

Freeze-Extraction is a suitable, easy to scale-up method for developing porous polymer structures without residual solvent problem [13]. It is a thermally induced phase separation technique, in which open, interconnected pores are formed via extraction of the frozen solvent (such as dioxane) from frozen polymer solution via a non-solvent (such as ethanol) for the polymer. However, the scaffolds prepared by this method did not have suitable mechanical properties for use in load bearing tissues [14]. But in our case, use of a robust titanium template would ensure the mechanical stability of a full hybrid implant. Also presence of the titanium template allows adjustment of the procedure in a way to obtain porosity gradients without the need to compensate for the associated strength loss.

Four main areas can be classified where it is beneficial to have pore gradients : 1) When there is a need to have different cell types which are in contact but yet spatially separated; [15], [16] 2) When the two parts of an organ or a junction (such as osteochondral junction) necessitates different properties; [17], [18] 3) When there is a need to control the migration rate of cells in a given direction; [19] 4) When it is important to retain cells in a spatial organization, for example to prevent restenosis related to smooth muscle cell movement in blood vessels while keeping the scaffold connected with the rest of the system for fluid and nutrient flow [20].

Here, we define production of an inter-connective, porous biodegradable polymer filling of the macroporous titanium tracheal implants for developing a system that enables fine control of different modes of cell movement in different areas of the implant. The final aim is to prevent fibroblast movement into the lumen for short term and at the same time to provide a suitable surface for the growth and migration of epithelial cells in vivo which are crucial for the functionality of composite tissues. By hindering the movement of the fibroblast, formation of epithelial layer will be promoted. Porous polymer filling was produced by successive use of thermally induced phase separation techniques within molds that directs the process hence permitting formation of pore gradients. A porosity gradient starting at the bottom of the material with macro open pores and turning into micropores that are large enough for cell infiltration was achieved. Then, in the upper part of the materials, these pores turns into lower microlevel ones which would obstruct cellular movement. This would permit fibroblasts to move within the scaffold but it would impede their migration to the upper surface since the average pore size on that face is lower than their size. On top of this structure a thin layer of polymer film either smooth or with nanolevel pores to induce 2D epithelial cell migration was also added.

## Results

**Figure 2:**
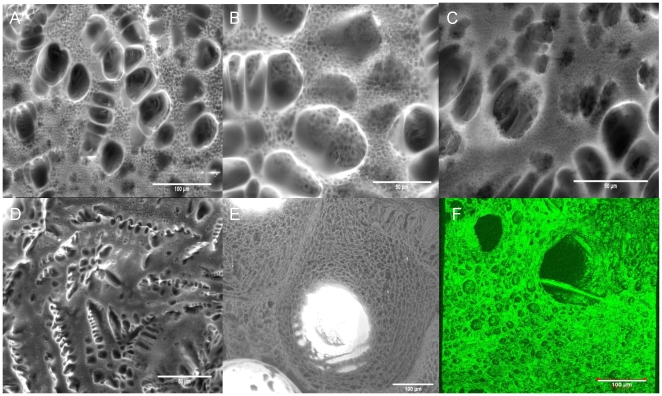
Pore structure of the hybrid implants. a–c) ESEM images of the back surface of the Titanium/PLLA hybrid prepared by 6% PLLA solution with a pre-gelling step at room temperature; distribution of small pores in between big pores due to the pregelling step is visible. d) Pore structure of the constructs when only PLLA was used. e) Coverage of the titanium beads with porous polymer filling f) 3D reconstruction of the back surface of the Titanium/PLLA hybrid obtained by confocal laser scanning microscopy.

The procedure to produce the pore gradient based on two steps. First, the pore gradient between the back and front surfaces was established by porous PLLA formation via freeze-extraction within a confining Teflon mold. The back surface is in direct contact with the extraction medium whereas the front surface is in contact with the Teflon mold which directs the movement of the extraction fluid. At the back, freeze-extraction process produced pores that are big enough to allow cell movement (Figure 2). The shape and the distribution of the pores were dependent on the polymer concentration and the cooling regime. For example a conventional open, interconnected pore structure can be achieved with lower concentrations (4%) and with a faster cooling regime. On the other hand, a high concentration of PLLA (6%) with a pre-gelation (incubation at room temperature) treatment produced a double-porous structure due to the phase separation within the gel body in which smaller pores are interspersed within the walls of bigger pores (Figure 2a–c; 2e). The morphology of the pores were quite similar to those obtained in the absence of Titanium and the Titanium body was totally engulfed by the polymer network (Figure 2e,f).

**Figure 3:**
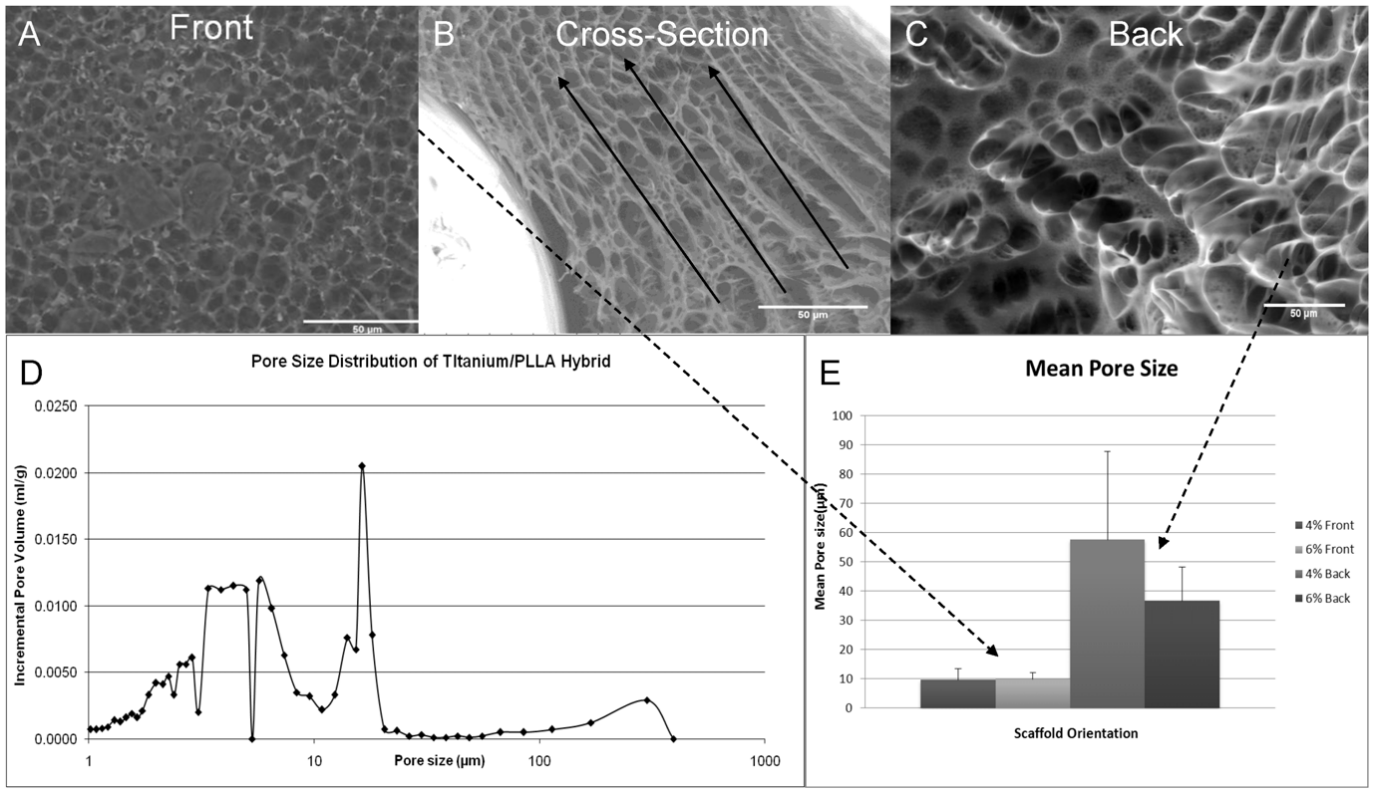
Pore gradient. a) Front surface of the hybrid implant with smaller pores. b) Cross-section of the hybrid implant shows the decrease in the pore size in the direction of ethanol in-flow which causes the pore gradient formation c) Back surface of the hybrid implant with bigger pores. d) Pore size distribution for the Titanium/PLLA hybrid; the distribution demonstrated the hierarchical gradient(6% PLLA). e) Difference in mean pore size between two surfaces was consistent in different PLLA concentrations where the front surface is less porous in both cases.

Analysis of the cross-sections of the PLLA impregnated titanium showed the decrease in the pore size along the cross-section, in which the pores are interconnected. Fluid movement can be observed in the shape of the pores (Figure 3b). Pore size distribution analysis of the filling shows a multi modal structure due to the preparation conditions (Figure 3 a,c). The difference in the porosity of the both side of the implant was significant which was determined by mercury porosimetry and image analysis (Figure 3 d–e). Mercury porosimetry results showed a 43% porosity for the hybrid system after PLLA impregnation with a primary average porosity of 5.5 µm and a secondary average of 16.4 µm. Moreover there was a distinct peak in the pore size distribution close to the values measured from SEM images which accounts for the highly porous area in the back side.

**Figure 4:**
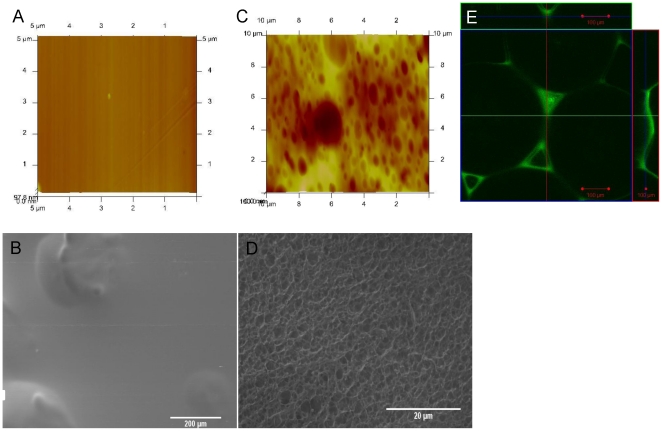
Surface nanoporous film layer. AFM and SEM images of a–b) solvent cast PLLA film and c–d) nanoporous top layer. A defined surface was obtained with both methods on top of Titanium/PLLA hybrid system. e) Confocal images of PLLA solvent casted on empty Titanium scaffolds. Solvent casting results in uniform PLLA distribution between the bead while the top layer follows the contour of the beads, whereas after freeze-extraction and film formation; a thin layer of polymer (∼4 µm) was on top of a body of porous PLLA covering the titanium beads.

In the second step, to improve the range of the gradient even further for reasons such as designing a surface with a pore size less than that of epithelial cells (less than 5 µm) and of suitable roughness (<1 µm) [21] for their successful culture, a film formation process on the surface is devised. Films are either formed by conventional solvent casting (non-porous) on the Titanium/PLLA hybrids or by an additional pore formation step to induce surface porosity. A dilute solution of PLLA (1%) in chloroform is applied on to the top surface of the structure for solvent-casting (Figure 4a, b) and in the second case, to induce pore formation, the hybrid is then immersed into ethanol. This step was done immediately after the addition of the PLLA solution and it would cause local precipitation of PLLA and thus pore formation in the film body (Figure 4 c, d). AFM images showed that the obtained surface had a surface average pore size of 836 nm with a wide range of pore size distribution (lower 110 nm, upper 2.9 µm). This porous surface had a higher roughness (Ra = 54.9±26.5 nm) compared to the solvent cast PLLA (Ra = 4.6±2.8 nm) films but still its surface roughness was below the limit of 1 µm necessary for epithelial cell culture. When the implant was only filled with polymer (no pore forming steps) formed layer followed the contours of the titanium beads (Figure 4e).

**Figure 5:**
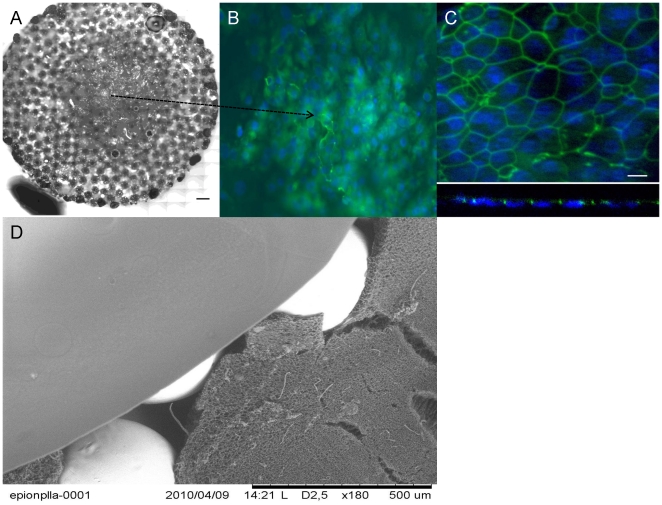
Overall structure of the hybrid implant and epithelialization. a) Collation of a full surface of a circular PLLA/Ti hybrid implant, seeded with freshly isolated Human respiratory epithelial cells isolated from nasal polyps(Scale bar: 500 µm). c) DAPI and anti-ZO1 stainings showed monolayer formation on the hybrid implant surface (Day 19). c) Confocal images on x–y plane and Z-section confirms the development of strong cell-cell contacts. d) SEM image of the overall hybrid structure, showing the titanium beads, the micropous body and the top film layer.

**Figure 6:**
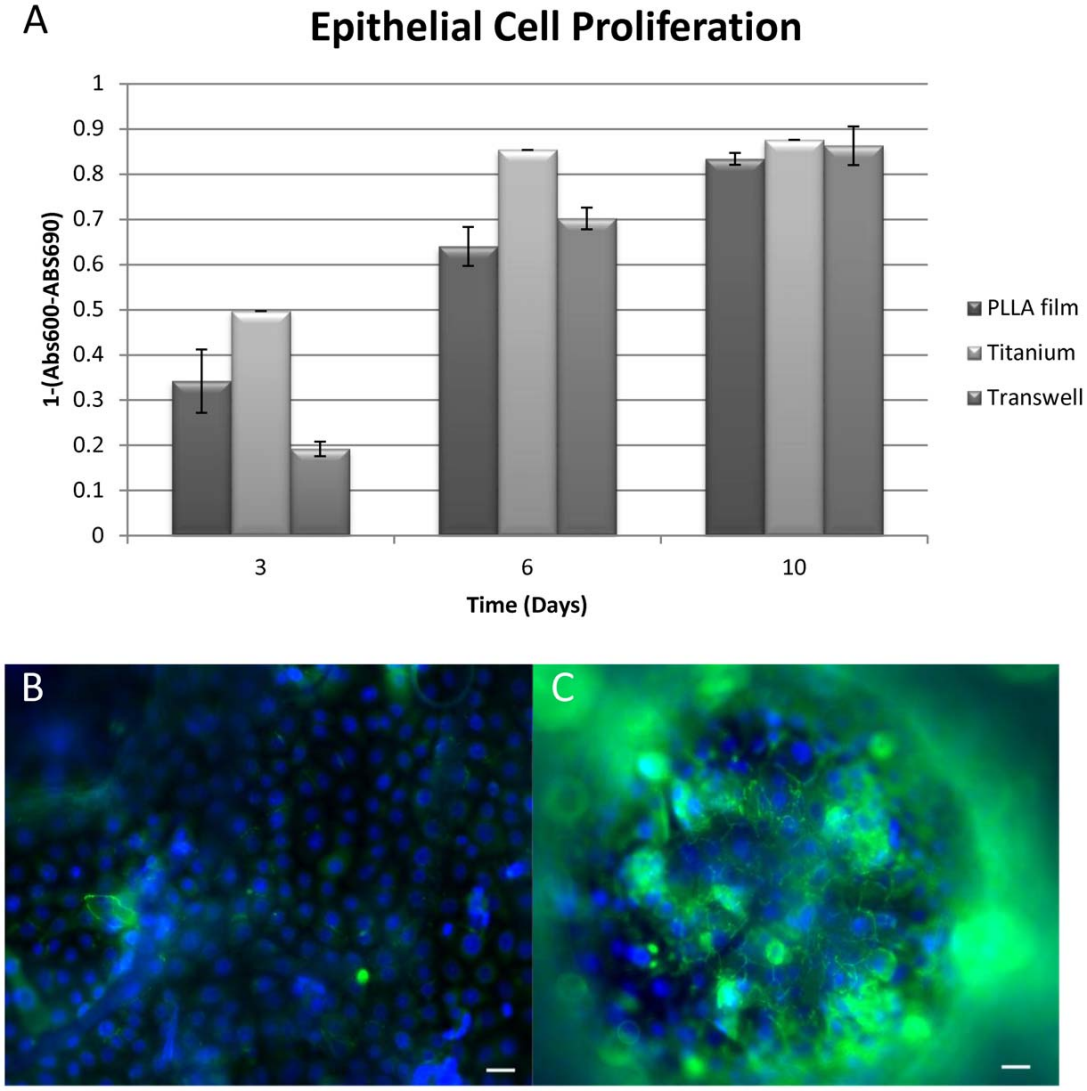
Epithelial cell proliferation and positioning. a) Human respiratory epithelium growth on transwell (positive control, hybrid implant with a nanoporous PLLA film and hybrid implant without a film b)presence of the film layer kept the epithelium on the same level for promoting the monolayer formation c) whereas in the absence of the film, epithelial cells grew on different levels (Scale bars: 10 µm).

Our tests with human respiratory epithelial cells also showed that nanoporous surface was suitable for their culture. The respiratory epithelial cells were able to proliferate and form cell to cell contacts on the hybrid system (Figure 5 a, b). Cellular proliferation on the porous film layers was comparable to that of the transwell membrane, which is the established culturing substrate for these cells (Figure 6a). However presence of the film layer helped the definition of the growth surface for the epithelial cells (Figure 6b), i.e cells stayed on the film layer. On the other hand, when seeded to scaffolds without any film layer the epithelial cells attach to the implant but cannot form a layer and were observed dissipated into the depth of the scaffold (Figure 6c). It was possible to observe the confluent layers via the macropores between the titanium beads by phase contrast microscopy (Figure S1).

**Figure 7:**
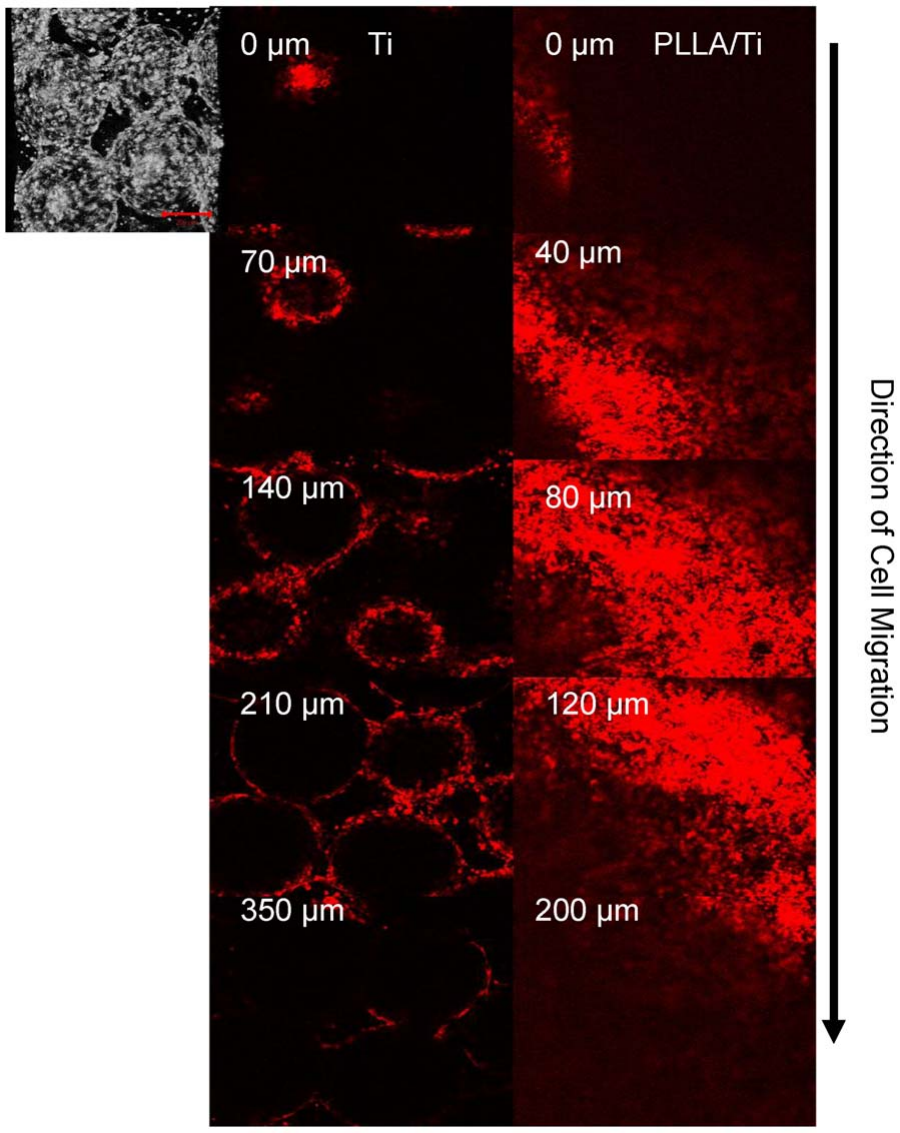
Vertical movement of 3T3 cells in Ti and Ti/PLLA implants. Distribution of 3T3 fibroblast after 7 day of culture from back to front for (Left hand side) Titanium Titanium/PLLA hybrid (Right hand side). Last images shows the depth through the scaffold where the signal (cell number) decreases significantly (∼350 µm and 200 µm respectively). Cells were able to move towards the front surface of the implant for both scaffold but the number of cells significantly decreased towards the front; where the migration degree was much higher in titanium scaffolds.

**Figure 8:**
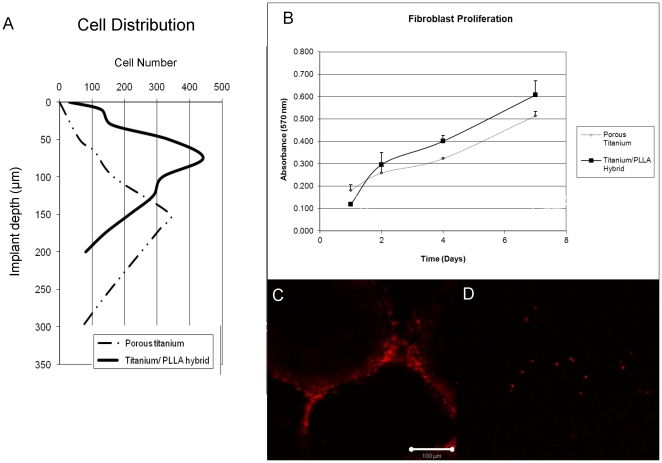
3T3 proliferation and the ability to reach to the other face of the implant. a) Depth of cell movement in Titanium only and Titanium/PLLA hybrids, cells moved considerably deeper in titanium only implants. The point were there was a significant drop in the signal (cell number) notified the final depth; b) MTT assay results for 3T3 proliferation: overall cell number was higher in PLLA/titanium hybrids due to decreased migration and the availability of more niches to proliferate; c) Confocal images of the front surfaces of the implants. A significant number of cells has reached the face of titanium only implants after 7 days of culture d) whereas only occasional cells can be observed in the case of Titanium/PLLA hybrids.

To test the hypothesis that the addition of the PLLA filling can impede cell movement, an aggressive fibroblastic cell line (NIH-3T3) [22] was seeded both on empty titanium and titanium/PLLA hybrids after cell surface was labeled with a non-toxic fluorescent marker (PKH26). The hybrids were placed on culture inserts face-down and the cells were seeded from the back. After 1 week of culture, cell migration was observed with confocal microscopy and the depth of the cell movement was determined (Figure 7). Cells moved considerably deeper into the titanium only implants (350 µm compared to 200 µm in titanium/PLLA hybrids) and the depth where the highest number of cells was present was also deeper (∼150 µm compared to ∼100 µm) (Figure 8a). The proliferation assays showed that cells grew more on PLLA/titanium hybrid compared to pure titanium, (Figure 8b). Moreover, the number of cells at the bottom layer of the implant was significantly less on Titanium/PLLA hybrids compared to pure titanium which showed that even though movement of the cells through PLLA is possible, it is slower compared to empty titanium (Figure 8 c,d).

## Discussion

In the area of trachea tissue engineering, there are two main challenges that needs to be overcome. First one is to obtain a collapse-resistant tubular structure tube that would enable the passage of air and the second one is the coverage of the inner surface of this tube with functional respiratory epithelium. In the body, the collapse resistance was provided by c-shaped cartilage rings, thus a considerable part of tracheal replacement efforts is focused on regeneration of this cartilaginous part [23]. However, the only function of these rings is the prevention of the tube's collapse, so the main requirements of the replacement are purely mechanical. But, the necessity of the epithelial layer inside imposes a vascular support beneath, thus the integration of the implant with the vasculature of the body becomes an important determinant [24]. The open porous structure of titanium is excellent for tissue ingrowth, but it lacks the ability to control the extent of the ingrowth. The design presented in this study provides the means to control the incoming fibroblast and inflammatory cell movement with a biodegradable scaffold, which would provide the necessary time for the migration of the epithelial cells from the anastomosis sites [25]. For obtaining a defined porosity gradient in the polymer body, the extraction process has been controlled by directing the inflow of the ethanol with a Teflon mold which resulted in a more porous back surface and a less porous front surface. This structure was clearly apparent in SEM images and results in slowing down of fibroblast movement. Moreover, in the double porous structure obtained the smaller pores would retard the movement of fibroblastic cells but provide large scale fluid flow thus enabling the nurturing of the cells while they are moving.

The ability of epithelial cells to migrate and to form a pseudostratified epithelium on the surface of the implant is limited and generally full epithelialization is never observed [26]. Cognizant of the necessity of seeding epithelial cells prior or after implantation, the current design contains a nanoporous structure providing a feasible surface for epithelial cell migration and culture. The nanoporosity would not hinder the movement of the epithelial cells, but can improve the degradation and finally would provide the route for vascularization of the top layer. Epithelial cells were able to grow on this surface and form cell-cell contacts on their way to forming an epithelial barrier. The next step in this aspect of the study is to differentiate the epithelial cells by air/liquid interface culture [27].

Change of the pore structure not only affected the cell movement but also had a slight effect on the proliferation: cells in the hybrid scaffold proliferated more. This might be due to the fact that the slowing down of cell movement had triggered proliferation or due to the increased amount of accessible surface area due to the presence of polymer. The open pores of the empty titanium are too large for cells to cover immediately, which would also explain the difference.

The next step would be testing the current system under in vivo conditions, to see whether it will hinder the movement of fibroblasts and decrease restenosis while providing enough time for slower epithelial cells to populate the inner lumen surface. This would dramatically improve the functionality of the implant. The porous structure would allow wide scale integration of the implant, as the cells can go through all the thickness of the implant over long implantation periods (together with the effect of polymer degradation); but since the inner surface would facilitate epithelialization this would not result in restenosis. This system might be further used in other target areas for cell separation too [28]. This design is currently being tested in new Zealand rabbits as tracheal implants.

## Materials and Methods

### Pore gradient formation within titanium implants

Macroporous titanium implants of 2 mm thickness and 11 mm diameter formed of medical grade Titanium beads with an open pore structure were provided by Protip (Strasbourg, France) and laser cutting by IREPA laser (Illkirch, France). 4% or 6% of PLLA (Sigma-Aldrich) solution was prepared in Dioxane/Water binary mixture (v∶v 87/13%) and then heated to 60°C up until a homogenous solution was obtained [29]. The hot solution was poured into the titanium implants via precision glass syringes in a two-piece custom-made Teflon mold up until all the pores are filled and the samples were either directly frozen or incubated at room temperature for 30 minutes before the freezing step. In this setting one face of the implant faces the Teflon which restricts the movement of extraction liquid in one direction and also affects the freezing of the solution. Samples were frozen overnight at −80°C. The next day, solvent exchange was achieved by immersion of the samples in the mold into pre-chilled 80% Ethanol solution at −20°C overnight [30]. Samples were removed from the molds and air dried. The morphology of the final product was observed with environmental SEM (Hitachi TM100, Japan) and the average pore size distribution and overall porosity was determined with mercury porosimetry analysis. To quantify the pore size difference between the back and front faces of the implants images of both sides (n>6) were processed and analyzed by Image J (NIH, USA) in which at least 50 definite, unconnected pore structures were measured per image to obtain the average surface porosity for each surface.

### Non porous/Nanoporous film formation on PLLA/titanium hybrids

After formation of the Ti/PLLA hybrid, to form the nanoporous top layer , 1% PLLA solution was dissolved in a more volatile solvent (Chloroform (Merck)) and 20 µl of the solution was applied on to the surface of Titanium/PLLA hybrid with precision glass syringes and then immersed in pure ethanol to induce phase separation [31]. After incubation in the non-solvent samples were removed and air-dried. The surface morphology, roughness and porosity were determined with Atomic force microscopy (Veeco). Thickness of the film layer and the morphology of the underlying porous layer and the effect of film formation on the porosity of the bottom layer were determined by confocal microscopy (Zeiss LSM 510), by utilizing the autofluorescence of PLLA.

### Fibroblast Cell culture and Cell migration assay

NIH-3T3 fibroblast cell line was cultured in RPMI 1640 medium (Gibco) with 10% Foetal bovine serum and 1% Penicillin/Streptomycin under standard culture conditions [22]. Titanium/PLLA hybrid and titanium only implants were sterilized by 70% Ethanol for 2 hours and then washed with sterile PBS and placed into 11 mm transwell cell culture inserts (Transwell) in order to restrict the movement of the cells to vertical direction after seeding. Confluent cells were removed with Tryple express enzyme cocktail (invitrogen), counted with a a haemocytometer and then marked with PKH26 fluorescent red cell linker (Sigma Aldrich) according to the providers instructions. Marked cells were seeded onto the implants in transwell inserts at a concentration of 2×10^6^ cells/implant and fed both from top and bottom. Medium was changed twice a day and at day 7, samples were fixed with 3.7% paraformaldehyde and observed with confocal microscopy (n≥3). For each sample at least 12 stacks were analyzed between the top layer (where the cells were seeded) and the layer where when the signal got nearly undetectable. Cell number at each thresholded stack was determined by Image J particle analyzer tool with a manual follow-up for noise reduction. To determine cell proliferation, samples were seeded with 1×10^5^ cells/implant and then cell numbers were determined over a course of 1 week with MTT cell proliferation assay (Promega) and validated with TOX8 (Resazurin based) assay (Sigma-Aldrich) (n≥6).

### Human Respiratory Epithelium Cell Isolation and Culture

For respiratory epithelial cell culture, freshly removed human nasal polyps were used for cell isolation as described before [32]. The use of human tissues was authorized by the bioethical law 94–654 of the Public Health Code of France, with a written consent from the patients. The study has been approved by the French committee “Comité de Protection des Personnes” (CPP-Est III, Nancy), statement n° DC-2008-374. The specimens used were polyps that needed to be removed, no surgeries were performed with the specific aim of obtaining polyps for the described experiments. Isolated cells were grown on tissue culture plates upon confluency. Cells were then seeded on hybrid Titanium/PLLA implants in transwell inserts at a concentration of 5×10^4^ cells/implant. Cell proliferation was observed by TOX8 assay over a 10 days period of time (n≥3). Culture was stopped at several time points and cell-cell contact formation and cell coverage of implant surface was observed by phase-contrast microscopy and by fluorescence microscopy after DAPI nuclei staining and ZO-1 immunodetection. Briefly, cultures were fixed for 10 min at −20°C in precooled methanol. After a wash in PBS (Gibco), they were saturated for 2 h in PBS containing 3% (vol/vol) of Bovine Serum Albumin (BSA, Sigma) to prevent unspecific bindings, then incubated overnight at 4°C with mouse anti-ZO-1 antibodies diluted at 1/100 in PBS/BSA 1% (Zymed). After 3 washes in PBS under gentle agitation, cultures were incubated with Alexe Fluor®-coupled goat anti-mouse IgG (Molecular Probe) at 1/200 in PBS for 1 h at room temperature (RT). After washes in PBS under gentle agitation, cell nuclei are finally stained with 4′,6-diamidino-2-phenylindole (DAPI; 1 mg/ml in PBS) for 15 min at RT, and cultures are mounted under coverslip in Aquapolymount antifading solution (Polysciences). Cultures were observed under an AxioImager fluorescence microscope (Zeiss) equipped with an Apotome device. Images were recorded with a CCD video camera (Coolsnap, Roper Scientific) at 57 successive z levels (1 µm between each z level) at ×20 magnification.

### Statistical Analysis

For determination of statistical significance Student's t-test was utilized for comparison of two conditions with significance level was set as p≤0.05, for tests involving more than two conditions ANOVA tests were performed with the same statistical significance level and Tukey's honest significance test as follow-up.

## Supporting Information

Figure S1 **Confluent Epithelial Cells on the film layer.** Phase-contast images of confluent human respiratory epithelial cells as observed in the film areas which lie on the macropores of the titanium body.
(TIF)
