# Supplementary figures and images for: Hybrid Titanium/Biodegradable Polymer Implants with an Hierarchical Pore Structure as a Means to Control Selective Cell Movement
Source: PLoS One. 2011 May 26;6(5):e20480. doi: 10.1371/journal.pone.0020480 (PMC3102721; doi:10.1371/journal.pone.0020480)

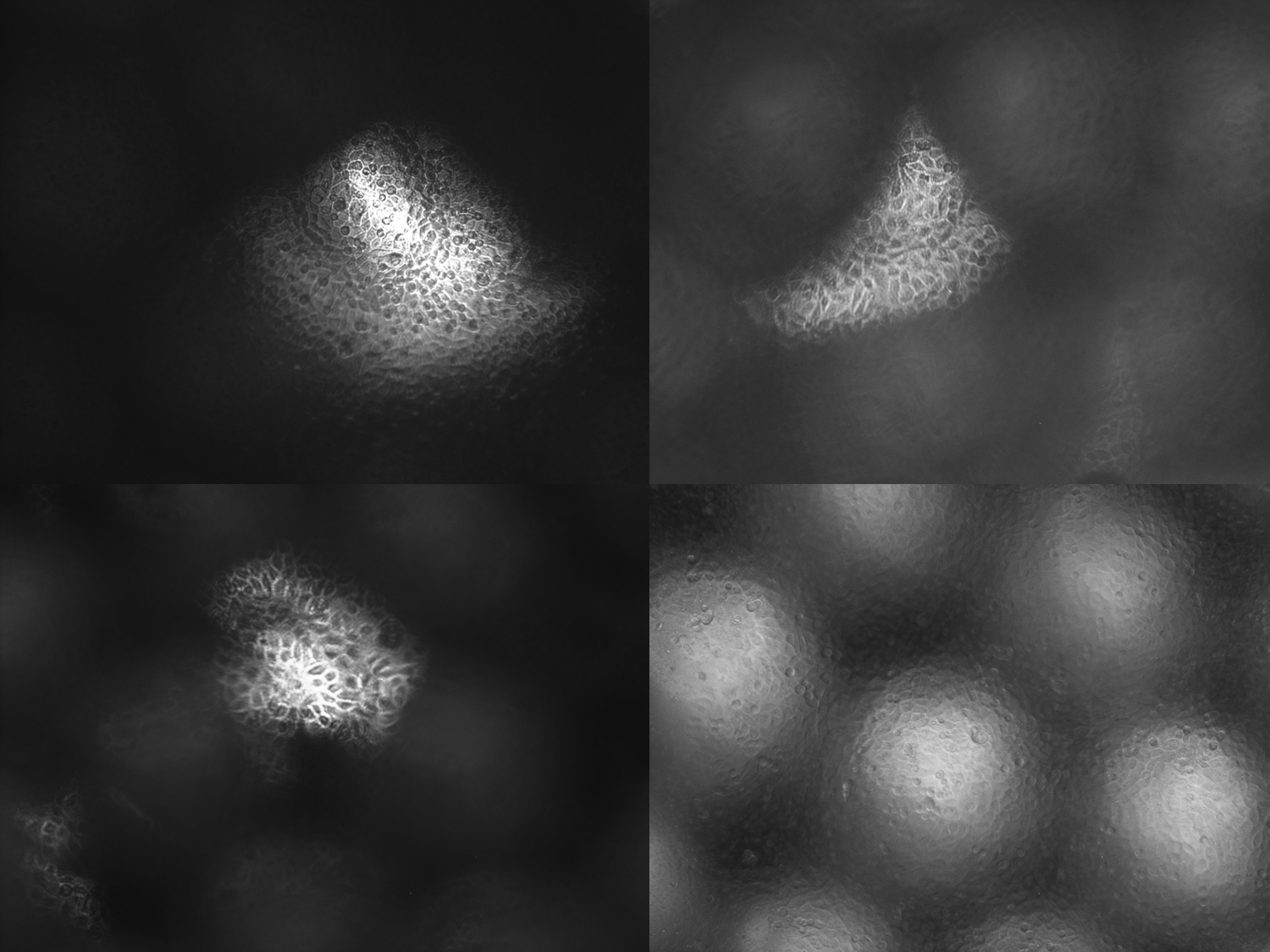

Supplement: Figure S1 — Confluent Epithelial Cells on the film layer. Phase-contast images of confluent human respiratory epithelial cells as observed in the film areas which lie on the macropores of the titanium body. (TIF) [file pone.0020480.s001.tif]
